# Clinical Outcomes in Patients Who Received a One-Time Aminoglycoside Dose for Extended-Spectrum Beta-Lactamase-Producing Enterobacterales or *Pseudomonas aeruginosa* Cystitis

**DOI:** 10.3390/antibiotics13060552

**Published:** 2024-06-13

**Authors:** Kelsey Bouwman, Melissa George

**Affiliations:** Department of Pharmacy, ECU Health Medical Center, Greenville, NC 27834, USA; melissa.george@ecuhealth.org

**Keywords:** urinary tract infections (UTIs), aminoglycosides, extended-spectrum beta-lactamase (ESBL), Enterobacterales, *Pseudomonas aeruginosa*

## Abstract

The Infectious Diseases Society of America (IDSA) recommends a single dose of an aminoglycoside for uncomplicated cystitis caused by extended-spectrum beta-lactamase (ESBL)-producing Enterobacterales (ESBL-E) and difficult-to-treat *Pseudomonas aeruginosa*. However, there is very little recent clinical evidence to support this recommendation. The objective of this study was to evaluate the safety and efficacy of a single-dose aminoglycoside for cystitis caused by ESBL-E or *Pseudomonas aeruginosa*. This was a multicenter, retrospective, cohort study. Patients who received ≥3 days of standard of care were compared to patients who received a one-time dose of an aminoglycoside with or without a short course of effective therapy before. The primary outcome was the rate of relapse defined as requiring escalation of antibiotics or starting new antibiotic therapy within 14 days after the completion of antibiotics. A total of 66 patients were included in this study, with 33 patients in each arm. There were more males and complicated cystitis patients in the standard-of-care group. There was no difference found in the rate of relapse. The length of stay was significantly shorter in the aminoglycoside group (4.5 ± 4.4 days vs. 14.1 ± 10.1 days, *p* < 0.0001). A one-time dose of an aminoglycoside did not increase the risk of relapse and was associated with a shorter length of stay when used to treat cystitis caused by ESBL-E or *Pseudomonas aeruginosa*.

## 1. Introduction

The number of hospitalizations due to urinary tract infections (UTIs) as well as the rate of extended-spectrum beta-lactamase (ESBL)-producing Enterobacterales (ESBL-E) and multi-drug resistant *Pseudomonas aeruginosa* urinary pathogens grew rapidly from 2000 to 2009 [1]. Drug resistance in patients with UTIs has been associated with increased healthcare costs and prolonged hospital length of stay [1]. Additionally, many of the oral antibiotics that treat ESBL-E and *Pseudomonas aeruginosa* cystitis have limitations that may preclude their use such as resistance, allergies, and side effects.

First line therapy for ESBL-E uncomplicated cystitis includes nitrofurantoin and sulfamethoxazole/trimethoprim (TMP-SMX) with alternative oral options being fluoroquinolones and fosfomycin [2]. Nitrofurantoin has evidence to support its efficacy in uncomplicated cystitis including for ESBL-E, but its use should be avoided in elderly adults and patients with impaired renal function due to the increased risk of adverse events in these patients [3,4,5]. In regard to TMP-SMX, there are reports of *Escherichia coli* becoming increasingly resistant [6]. Additionally, it is not an option for patients with a sulfa allergy, and it has many side effects, including hyperkalemia, which can be especially problematic in patients with poor renal function or patients receiving concomitant renin–angiotensin system-acting agents, both of which are commonly encountered in hospitalized patients [7]. Lastly, fosfomycin is only effective for Gram-negative UTIs caused by *Escherichia coli* due to the presence of the fosA gene in many other organisms, making them intrinsically resistant [8]. Oral options for *Pseudomonas aeruginosa* are limited to fluoroquinolones, which have increasing resistance, a large number of side effects such as QT prolongation and tendon rupture, and multiple drug interactions [2,9]. Therefore, oral options can be extremely limited for these highly resistant organisms.

Historically, patients who could not receive oral therapy for cystitis would receive a 3-to-7-day course of intravenous (IV) antibiotic therapy [9,10,11]. Patients with ESBL-E commonly receive ertapenem or meropenem, and patients with *Pseudomonas aeruginosa* commonly receive cefepime, piperacillin/tazobactam, or meropenem [2]. Because of this, patients may require outpatient parenteral antibiotic therapy with or without a central line and/or can have prolonged hospitalizations. Additionally, due to their broad spectrum of activity, it is preferred to reserve these antibiotics for severe infections. This makes a single dose of an aminoglycoside an appealing option in patients with no oral options for uncomplicated cystitis. The Infectious Diseases Society of America (IDSA) recommends a single dose of an aminoglycoside as an alternative option for uncomplicated cystitis due to ESBL-E and a first-line recommendation for uncomplicated cystitis due to difficult-to-treat *Pseudomonas aeruginosa* [2]. Theoretically, due to their post-antibiotic effect, a single dose of an aminoglycoside would also be effective for completing therapy in patients with complicated cystitis when given following a 3-to-4-day course of appropriate therapy to total 7 days. However, there is very little recent clinical evidence to support the use of a single dose of an aminoglycoside in cystitis. The primary study investigating this treatment strategy was a systematic review conducted by Goodlet et al. in 2018, and it found an equal number of relapses and reinfections [12]. 

The purpose of this study was to evaluate the safety and clinical efficacy of a single dose of an aminoglycoside for cystitis caused by ESBL-E or *Pseudomonas aeruginosa* in adult patients. If safe and effective, a one-time dose of an aminoglycoside could prevent issues with adherence and decrease hospital length of stay.

## 2. Results

**Figure 1:**
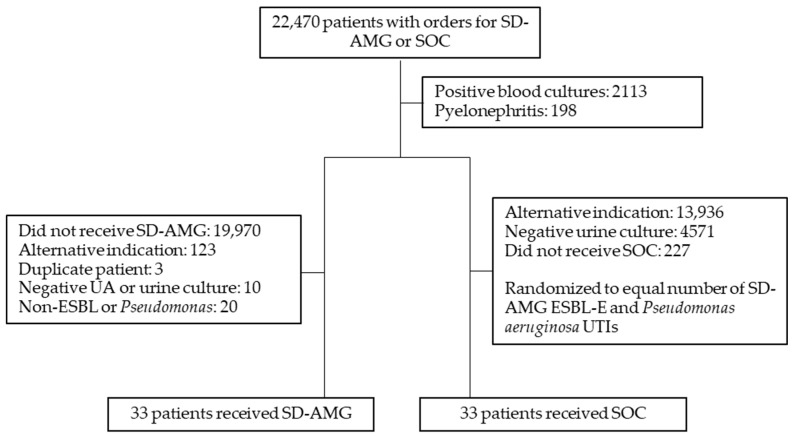
Patient selection. SD-AMG: single-dose aminoglycoside; SOC: standard of care.

A total of 66 patients were included in this study, with 33 patients in each study arm (Figure 1). Demographics were similar between the two groups with a median age of 74–78 years old and most patients being either white or African American. There were more male patients (24.2% vs. 57.6%) and more patients with complicated cystitis (33.3% vs. 72.7%) in the standard-of-care group compared to the aminoglycoside group. There were also more patients in the intensive care unit (ICU) in the standard-of-care group (3% vs. 21.2%), whereas the aminoglycoside group had more patients being treated outpatient (39.4% vs. 9.1%). The most common pathogens in the ESBL Enterobacterales group were *Klebsiella pneumoniae* and *Escherichia coli*. (Table 1).

The most common aminoglycoside used was gentamicin. Effective antibiotics prior to receiving a one-time dose of an aminoglycoside were given to 15/33 (45%) of patients for an average of 3.2 ± 1.4 days. The average doses of the aminoglycosides were 5.1 ± 1.2 mg/kg for gentamicin, 4.6 ± 1.6 mg/kg for tobramycin, and 13 ± 4.2 mg/kg for amikacin. These doses are similar to those recommended for this indication, 5 mg/kg for gentamicin and tobramycin and 15 mg/kg for amikacin [2]. In the standard-of-care group, the most common antibiotic used was ertapenem for ESBL-E and piperacillin/tazobactam for *Pseudomonas aeruginosa*. The average duration of therapy in the standard-of-care group was 6.91 ± 2.35 days. All doses were assessed based on weight and renal function and found to be appropriate based on our institution’s recommendations (Table 2). 

### 2.1. Primary Outcome

There was no difference in relapse when comparing a single dose of an aminoglycoside to the standard of care (1/33, 3.03% vs. 3/33, 9.09%; 95% CI 0.03–3.04; *p* = 0.6), as seen in Table 3. The pre-specified subgroup analysis demonstrated no difference in the primary endpoint for patients with ESBL-E (0.06% in both groups), *Pseudomonas aeruginosa* (0% in the aminoglycoside group vs. 3.03% in the SOC group), complicated cystitis (0.07% in the aminoglycoside group vs. 0.08% in the SOC group), and uncomplicated cystitis (0% in the aminoglycoside group vs. 0.11% in the SOC group) (Table 4).

### 2.2. Secondary Outcomes

There was no difference in the secondary endpoints of the readmission rate for UTI (0% vs. 3.03%), and no significant development of aminoglycoside resistance was observed. There was a significant difference found in the length of stay, with it being 4.5 ± 4.4 days in the aminoglycoside group and 14.1 ± 10.1 days in the standard-of-care group (*p* < 0.001). There was no difference in the safety outcomes of AKI and ototoxicity (Table 3). 

## 3. Discussion

In this retrospective study, a one-time dose of an aminoglycoside did not increase the risk of relapse or readmission when compared with the standard of care at 14 days after the completion of antibiotics for ESBL-E or *Pseudomonas aeruginosa* cystitis. A one-time dose of an aminoglycoside was associated with a decrease in the length of stay. However, the length of stay may have been confounded by additional comorbidities considering the larger number of patients with complicated cystitis and patients requiring ICU-level of care at baseline in the standard-of-care group. There were limited data available to assess the development of resistance and safety outcomes, but there were no increased risks of nephrotoxicity, ototoxicity, or aminoglycoside resistance found in this study. 

Aminoglycosides were historically used to treat UTIs, but they eventually fell out of favor due to their side effects of ototoxicity and nephrotoxicity [12]. However, they are excreted in high concentrations in the urine. The concentration of aminoglycosides in the urine can exceed their plasma concentrations by up to 100-fold within an h of parenteral administration, and these concentrations remain above therapeutic levels longer than 72 h for most uropathogens [12]. Additionally, a single dose of an aminoglycoside has been reported to contain lower rates of nephrotoxicity and vestibular toxicity compared to a conventional 7-day course of aminoglycosides [13]. 

Currently, there are limited data available to support the IDSA’s recommendation for a single dose of an aminoglycoside to treat cystitis. The primary study investigating this was conducted by Goodlet et al., who completed a systematic review in 2018 to review the efficacy of a single dose of an aminoglycoside for the treatment of UTIs [12]. Thirteen studies were included in their systematic review. They found a microbiologic cure rate of 94.5% and a 30-day recurrence rate of 19%, with an equal number of relapses and reinfections. Only 0.5% of patients experienced side effects such as nephrotoxicity, vestibular toxicity, discomfort at injection site, and transient paresthesia. This systematic review did have several limitations, such as the fact that 53.8% of these studies only included children, many of the studies were completed in the 1980s and 1990s, and the majority of comparator antibiotics are no longer commonly used as first-line treatment for UTIs. We chose not to assess for microbiologic cure, which was determined for 11 out of 13 studies in the systematic review, since it is not standard practice to confirm cure with follow-up urine cultures. Our study also focused specifically on cystitis caused by highly resistant organisms. However, the results of our study in adult patients are consistent with the systematic review, as we also found no difference in the rate of relapse, with a very small number of side effects. 

There has been one additional prospective cohort study evaluating the safety and efficacy of single-dose aminoglycosides for the treatment of complicated cystitis for 13 patients in the emergency department. In total, 85% of these patients had no oral antibiotic option for discharge based on prior cultures in the past year. Only three patients (23%) met their primary endpoint, which was clinical or microbiological failure at 14 days, defined as unresolved UTI symptoms, new symptoms, death, or having a urine culture positive for the same pathogen identified on study entry. Most patients who experienced failure had multiple complicating factors [14]. Since their primary outcome was slightly different than ours and there is a noticeable difference in the sample sizes, it is somewhat difficult to make more direct comparisons between their study results and ours. However, these results are consistent with our overall study conclusions, demonstrating the safety and efficacy of single-dose aminoglycosides for complicated cystitis. Most patients in our study received a short course of effective antibiotic therapy for 3 days prior to being given a single dose of an aminoglycoside, so our patients had a longer total duration of therapy.

In a patient otherwise stable for discharge, a single dose of an aminoglycoside can complete therapy for ESBL-E or *Pseudomonas aeruginosa* cystitis. When no oral options are available, a one-time dose of an aminoglycoside can prevent the continued need for broad-spectrum IV antibiotics for these highly resistant organisms. They may also be able to assist in avoiding prolonged hospitalizations and discharging patients with central lines. Based on the results of this study, the use of a single dose of an aminoglycoside did not have worse outcomes compared to the standard of care in regard to relapse or readmission rates. However, it is important that patients are clinically stable if a one-time dose of an aminoglycoside is used to treat cystitis, as the majority of patients in our aminoglycoside group were outpatients or admitted to a medicine unit. Additionally, patients with complicated cystitis should receive effective therapy for at least 3 days before a single dose of an aminoglycoside in order to complete a total duration of 5 to 7 days of antibiotics, consistent with the current duration recommended by the IDSA guidelines [10]. 

This study included several limitations. This was a retrospective study, and it included a small number of patients, resulting in this study being underpowered. We also excluded patients with bacteremia and pyelonephritis, so the results of this study cannot be applied to these patients. The study groups were not completely balanced, with more complicated cystitis patients and patients requiring ICU-level care in the standard-of-care group, which likely contributed to the longer length of stay. However, no difference in our primary endpoint was found in the subgroup analysis of complicated vs. uncomplicated patients. Both inpatient and outpatient antibiotics were reviewed regarding our primary endpoint, but we did not have access to outpatient information on patients more than a year out from their index infection or who were admitted to another health system. This could have resulted in missing patients with relapses or readmissions. Information was collected on whether patients presented with typical symptoms of a UTI, but it was not utilized as an inclusion or exclusion criterion, which could have led to including patients with asymptomatic bacteriuria. We attempted to limit the impact of patients colonized with these organisms by only including each patient’s first admission within this study’s time frame and patients with a urinalysis with pyuria or 3+ leukocyte esterases. 

During the process of this study, the Clinical and Laboratory Standards Institute (CLSI) lowered the susceptibility breakpoints of all aminoglycosides for Enterobacterales; specifically, the breakpoint for amikacin was lowered by 4-fold. All data were collected before these changes, and our system’s susceptibility panel did not report minimum inhibitory concentrations (MICs) as low as the new breakpoints. Aminoglycosides likely remain effective for ESBL-E cystitis since they are highly concentrated in the urine. However, in most patients who received amikacin for ESBL-E cystitis, it was the only aminoglycoside susceptible. The change in amikacin’s breakpoint will likely reduce the number of isolates interpreted as susceptible in the future. Regarding *Pseudomonas aeruginosa*, the breakpoint of amikacin remained the same, but it should only be considered effective for *Pseudomonas aeruginosa* in the urine. The breakpoint of tobramycin was lowered, and gentamicin is now classified as intrinsically resistant to *Pseudomonas aeruginosa*. Gentamicin was chosen to treat cystitis in 12 out of 18 patients who grew *Pseudomonas aeruginosa* in this study, so it is possible that gentamicin was not effective for these patients. However, given the low rate of relapse overall in our study, these results may either suggest otherwise or may suggest that these patients did not have a true UTI to begin with. Additionally, one could consider the phenomenon known as the “90–60 rule” being at play, which suggests that 60% of resistant isolates still have successful clinical outcomes [15]. In light of these changes, tobramycin and amikacin can still be considered effective for cystitis based on their susceptibilities, but gentamicin should no longer be recommended for *Pseudomonas aeruginosa*. 

This retrospective study of patients with cystitis caused by ESBL-E or *Pseudomonas aeruginosa* found that a one-time dose of an aminoglycoside did not increase the risk of relapse at 14 days after the completion of antibiotic therapy and was associated with a shorter length of stay. While there are several limitations to this study, it adds to the small body of literature available to support a single dose of an aminoglycoside for cystitis in adult patients. A prospective study including a larger number of patients is needed to confirm these results and further support the recommendation of a one-time dose of an aminoglycoside for cystitis.

## 4. Materials and Methods

This retrospective, cohort study was performed at nine hospitals in the East Carolina University (ECU) Health system, which includes one academic medical center and eight community hospitals. Patients treated for ESBL-E or *Pseudomonas aeruginosa* cystitis from 1 January 2020 to 31 December 2022 were identified for analysis through medication administration records, and all data were obtained from electronic health records. Patients were included if they were ≥18 years old, had a urinalysis with WBC > 10/HPF or 3+ leukocyte esterase, had ESBL-E or *Pseudomonas aeruginosa* isolated from a urine culture, and received a one-time aminoglycoside dose or the standard of care with a selected indication of urinary tract infection on the antibiotic order. Only the first admission for ESBL-E or *Pseudomonas aeruginosa* cystitis within this study’s time frame was included for each patient. This study included both complicated and uncomplicated cystitis. Complicated cystitis was defined as being male, the presence of a urinary catheter, urinary tract obstruction, renal tract calculi, colovesical fistula, and/or uncontrolled diabetes. Patients were excluded if they had positive blood cultures, a diagnosis of pyelonephritis, had received <3 days of the standard-of-care therapy, or were able to receive oral therapy for their UTI. Baseline characteristics were collected through a review of electronic medical records and included age, weight, gender, race, history of kidney disease, and the level of care the patient was receiving at time of antibiotic administration (i.e., outpatient, medical service, ICU). Bacterial Identification and antimicrobial susceptibility tests were performed using the MALDI-TOF and MicroScan WalkAway automated systems. Susceptibility results were interpreted according to the CLSI 2022 guidelines. The dosing of all antibiotics was assessed by a pharmacist and deemed appropriate or inappropriate based on our institutional guidance depending on patient weight and renal function.

Patients who received ≥3 days of standard of care were compared to patients who received a one-time dose of an aminoglycoside with or without an effective short course of therapy before. The patients included could have received a single dose of IV amikacin, tobramycin, or gentamicin. The short course of effective antibiotics was defined as receiving IV antibiotics that the urinary isolate was susceptible to for a duration of 3–4 days prior to receiving a one-time dose of an aminoglycoside. This effective short course of therapy would allow patients in the aminoglycoside group with complicated cystitis to receive a total of 7 days of effective therapy. The standard of care was defined as a carbapenem (ertapenem or meropenem), cefepime, or piperacillin/tazobactam.

The primary endpoint was the rate of relapse, defined as requiring escalation of antibiotics or starting new antibiotic therapy within 14 days after the completion of antibiotics. Secondary endpoints included readmission rate within 14 days for UTI, development of aminoglycoside resistance, average aminoglycoside dose utilized, and length of stay. Development of aminoglycoside resistance was defined as a difference in aminoglycoside susceptibility on urine culture with the same pathogen and within 1 year of index infection if urine cultures were available. Safety endpoints included patient-reported ototoxicity and the incidence of acute kidney injury (AKI), defined as an increase in serum creatinine by ≥0.3 mg/dL within 48 h or a 1.5-fold increase from baseline. 

Descriptive statistics were performed for analysis. The rate of relapse, rate of readmission, and development of resistance were assessed with Fisher’s exact test. The average length of stay was assessed with the Mann–Whitney test. For the primary endpoint, a sub-analysis stratified by pathogen and complicated vs. uncomplicated cystitis was performed. A *p* value of ≤0.05 was considered to be statistically significant. It was calculated that a sample size of 150 patients in each group would be required to reach 80% power in order to detect a 5% reduction in the primary endpoint with a prespecified alpha of 0.05. All statistical tests were performed via GraphPad. 

## 5. Conclusions

A one-time dose of an aminoglycoside did not increase the risk of relapse at 14 days after the completion of antibiotic therapy for ESBL Enterobacterales and *Pseudomonas aeruginosa* cystitis and was associated with a shorter length of stay. However, the length of stay may have been confounded by differences in baseline characteristics. While larger, randomized clinical trials are needed to further evaluate this treatment strategy, our data suggest a one-time dose of an aminoglycoside is safe and effective for ESBL Enterobacterales and *Pseudomonas aeruginosa* cystitis.

## Figures and Tables

**Table 1:** Baseline characteristics.

	Aminoglycoside (n = 33)	SOC (n = 33)	*p* Value (95% CI)
Age (years); median (IQR)	78 (73.0–85.0)	74 (66.0–83.0)	0.20
Weight (kg); median (IQR)	68 (59.2–79.3)	81 (64.3–93.0)	0.08 (−1.2–18)
Male; n (%)	8 (24.2)	19 (57.6)	0.01 (0.2–0.8)
Race; n (%)			0.06 (0.3–1.0)
African American	6 (18.2)	14 (42.4)	
Hispanic or Latino	1 (3.0)	1 (3.0)	
White	26 (78.8)	17 (51.5)	
Unknown	0	1 (3.0)	
Level of care; n (%)			0.004
Outpatient	13 (39.4)	3 (9.1)	
Medicine service	19 (57.6)	23 (69.7)	
Intensive care unit	1 (3.0)	7 (21.2)	
History of kidney disease; n (%)	9 (27.3)	15 (45.5)	0.20 (0.9–2.7)
ESBL Enterobacterales; n (%)	16 (48.5)	16 (48.5)	
*Escherichia coli*	4 (12.1)	8 (24.2)	
*Klebsiella oxytoca*	2 (6.1)	0	
*Klebsiella pneumoniae*	10 (30.3)	7 (21.2)	
*Proteus mirabilis*	0	1 (3.0)	
*Pseudomonas aeruginosa*; n (%)	18 (54.5)	18 (54.5)	
Typical symptoms present *; n (%)	13 (39.4)	9 (27.3)	0.43 (0.5–1.2)
Complicated cystitis **; n (%)	11 (33.3)	24 (72.7)	0.01 (1.3–3.8)
Urinary catheter present; n (%)	8 (24.2)	10 (30.3)	0.85 (0.5–1.5)
Rate of catheter exchange; n (%)	8/8 (100)	9/10 (90)	

* Typical symptoms included dysuria, urinary frequency, and urinary urgency. ** Complicated cystitis defined as including the following characteristics: male, urinary catheter, obstruction, renal tract calculi, colovesical fistula, or uncontrolled diabetes.

**Table 2:** Antibiotic therapy.

Aminoglycoside; n (%)	
Amikacin	10/33 (30.3%)
Gentamicin	16/33 (48.5%)
Tobramycin	7/33 (21.2%)
Patients who received appropriate short course of antibiotics prior to aminoglycoside; n (%)	15/33 (45.5%)
Mean (SD) duration of appropriate short course of antibiotics prior to aminoglycoside (days)	3.2 ± 1.4
Mean (SD) aminoglycoside dose (mg/kg)	
Amikacin	13 ± 4.2
Gentamicin	5.1 ± 1.2
Tobramycin	4.6 ± 1.6
Standard-of-care antibiotics; n (%)	
Cefepime	8/33 (24.2%)
Ertapenem	14/33 (42.4%)
Meropenem	2/33 (6.1%)
Piperacillin/tazobactam	10/33 (30.3%)
Mean (SD) standard-of-care duration of therapy (days)	6.91 ± 2.35
Appropriate dose for standard-of-care antibiotics; n (%)	33/33 (100%)

**Table 3:** Outcomes.

	Aminoglycoside	SOC	*p* Value (95% CI)
Rate of relapse	1/33 (3.03%)	3/33 (9.09%)	0.613 (0.037–3.044)
Readmission rate	0/33 (0%)	1/33 (3.03%)	1.0
Average length of stay (days)	4.5 ± 4.4	14.1 ± 10.1	<0.0001
Development of aminoglycoside resistance	4/11 (36.4%)		
Safety			
AKI *	0/3	0/12	
Ototoxicity	0/33	0/33	

* AKI only assessed in patients with SCr available after therapy was completed.

**Table 4:** Rate of relapse subgroup analysis.

	Aminoglycoside	SOC	*p* Value (95% CI)
ESBL Enterobacterales	1/16 (0.06%)	1/16 (0.06%)	1.0 (0.07–14.7)
*Pseudomonas aeruginosa*	0/18 (0%)	2/18 (0.11%)	0.49
Complicated cystitis	1/13 (0.07%)	2/24 (0.08%)	1.0 (0.09–9.2)
Uncomplicated cystitis	0/20 (0%)	1/9 (0.11%)	0.31

## Data Availability

The raw data supporting the conclusions of this article will be made available by the authors on request.
